# Supplementary material for: Are carbon emissions trading and green financial instruments synergistic? -Comprehensive quantitative research based on content analysis
Source: PLoS One. 2024 Mar 7;19(3):e0298601. doi: 10.1371/journal.pone.0298601 (PMC10919601; doi:10.1371/journal.pone.0298601)
Supplement: S1 Data — (ZIP) [file pone.0298601.s001.zip › Data/地级市Z_F工作报告.zip!/║┌┴·╜¡/╣■╢√▒⌡╩╨/└┤╫╘╣1⁄2╓┌║┼í╛┬φ┐╦╩2╛▌═°í┐_2010.docx]

各位代表：

　　现在，我代表市人民政府，向大会作工作报告，请予审议，并请市政协各位委员提出意见。

　　2009年工作回顾

　　去年是极不寻常、极不平凡的一年。面对国际金融危机的冲击，在省委、省政府和市委的坚强领导下，市政府坚持以科学发展观为统领，攻坚克难，逆势奋进，全力保增长、保民生、保稳定，较好地完成了市十三届人大三次会议确定的各项任务。

　　经济平稳较快增长。全年实现地区生产总值3258.1亿元，在东北地区继沈阳、大连之后第三个进入3000亿元行列，比上年增长13%。完成财政一般预算总收入338.4亿元，增长20.6%，其中地方财政一般预算收入193.4亿元。完成全社会固定资产投资1892.1亿元，增长41.1%。实际使用外资6.05亿美元，增长11.4%；国内经济技术合作实际到位369.4亿元，增长25.3%。完成进出口总额36.9亿美元，增长1.3%。16项主要经济指标增幅在15个副省级城市比较中，有11项位次前移，其中投资、消费等6项指标增幅进入前三名。

　　民生状况继续改善。10大类100项惠民行动项目投入160亿元，为上年的2.7倍，是近年来投入最多、惠及群众最广的一年。城镇新增就业10.7万人，转移农村劳动力126.6万人，城镇登记失业率控制在3.04%以内。城镇人均可支配收入达到15887元，增长8.9%。农民人均纯收入达到6776元，增长13.7%。

　　社会环境平安和谐稳定。全年信访总件次下降5.2%，各类安全事故主要指标大幅下降，刑事案件万人发案率在15个副省级城市中保持最低，荣获“全国社会治安综合治理优秀城市”称号。

　　我们主要抓了以下工作：

　　——积极应对国际金融危机等不利因素影响，开创了经济回升向好新局面。注重思路引领。及时确定了“提升存量强实力、扩大内需增动力、深化改革激活力、自主创新挖潜力、发展金融添助力、坚定信心聚合力”的工作原则，推出了“抓项目建设扩大投资，抓促进消费搞活流通，抓优势产业以丰补歉，抓改革开放激发活力，抓结构调整加快转变，抓自主创新集成竞争，抓改善民生提高福祉，抓效能建设优化环境，保增长、促发展”的“八抓八保”务实举措，明确了“千方百计扩大投资，千方百计抓重点抓关键，千方百计培育和壮大新经济增长点，千方百计促进全民创业”的总体要求。实施政策推动。创造性地贯彻落实国家“一揽子”计划和省“组合拳”政策，因地制宜出台了工业企业贷款担保风险备付资金、工业保增长促发展34条、中小企业专项资金、对外贸易发展专项资金、保持房地产业健康发展20条、做好劳动保障工作18条等系列政策。这是近年来我市出台地方性经济政策最多、配套程度最高的一年。强化内需拉动。以“六个一百”为重点的“项目建设年”部署稳步实施，投资大幅增长，比三年前翻了一番。积极争取国家和省“一揽子”计划投资支持，共获得中央和省投资16.4亿元。认真落实“家电下乡”、“汽车、摩托车下乡”和汽车以旧换新政策，及时兑现补贴资金，大力推进“万村千乡市场工程”和社区商业“双进工程”，城乡市场供销两旺，实现社会消费品零售总额1507.9亿元，增长19.3%。着力培育和壮大旅游、房地产等12个新的经济增长点，实现了协同拉动、以丰补歉。旅游业实现总收入310.2亿元，增长26.2%，被评为“中国十大节庆城市”。房地产市场“暖春行动”成效显著，实现商品房销售额297.6亿元，增长33.8%；建筑业增加值增长13.7%。坚持产业带动。开展了“保重点、遏下滑、促增长”战役，力保重点行业发展不停滞、重点企业壮大不萎缩、粮食产量、农业效益和农民收入增长不下滑。工业经济迅速企稳回升，规模以上工业实现增加值581.7亿元，增长14.5%。工业效益大幅增长，实现利税创历史新高。农林牧渔业总产值实现724.1亿元，增长6.9%。粮食生产再创历史新高，总产量达226.2亿斤。

　　——注重打基础、攒后劲，全面协调可持续发展能力建设取得新进展。创新完善发展战略。把松花江开发保护利用作为城市发展的战略总纲，完成了沿江产业带发展规划及保护建设“万顷松江湿地、百里生态长廊”、“现代北国水城”、“江北科技创新城”、“哈南工业新城”等20余项重大规划编制任务，出台了《关于实施强县战略的指导意见》。扎实推进结构调整。加快发展装备制造、高新技术、食品、医药、化工5个优势主导产业，生物、新能源、新材料3个新兴战略产业，现代金融、物流、旅游、房地产4个服务业，三次产业增加值分别增长6.8%、13.3%和14.4%，装备制造、食品、医药和化工4大优势产业占规模以上工业增加值比重达83.2%，畜牧业占农林牧渔业比重提高到46.1%。投资结构优化，完成工业投资545.8亿元，增长56%，高于全市固定资产投资增幅14.9个百分点，其中生产性投资542.6亿元，占工业总投资的99.4%。完成民间投资965.3亿元，增长49.3%。产品结构得到调整，新增中国驰名商标4件，全市现有中国驰名商标总数达到17件。所有制结构发生积极变化，非公有制经济增加值增长14%，占地区生产总值比重达50.8%。切实加强城乡基础设施建设。完成城市基础设施投资664.7亿元，增长49.9%，地铁一期、松浦大桥、道路改造、城市堤防升级达标等重点工程进展顺利，磨盘山供水、西泉眼水库加固扩容、城市气源转换等工程全面完成。公路建设“三年决战”扎实推进，绕城高速公路东北段、黑大公路拉林经五常至省界段竣工通车，通乡（镇）、通村公路硬化率分别达到98%和92%。大力推进新区开发建设。群力新区资源利用和区域开发效益明显提升，成为新的投资热点和宜居新城区。哈西客站建设正式启动。集中整治城市卫生、交通、服务环境。清扫保洁、清冰雪作业市场化进程加快，主干街路大雪、中雪做到一日清完。综合整治城市交通秩序，实施单向通行道路182条，辟建停车泊位1.82万个，取缔交通违法行为73.6万件。优化行政服务环境，取消行政审批要件118个，减少审批环节812个，向区下放事权19项。全面加强生态建设。松花江流域水污染防治项目稳步推进，干流出境水主要污染指标下降3.4%。完成减排项目100个，“十一五”二氧化硫排放量和化学需氧量减排目标提前一年完成。新建国家级生态示范区2个、省级自然保护区1个，空气优良天数310天，首次达到国家环保模范城市标准，城区新植树木122.4万株，新增绿地2137.4公顷。完成造林绿化42.5万亩。农村环保工作扎实推进。

　　——大力推动改革创新，为应对危机保增长注入了新动力。推进体制创新。完成哈量集团与通用技术集团等战略重组，65户厂办大集体完成整体改制，市管国有及国有控股企业绩效考核实现全覆盖。公共卫生、公共交通、公共医疗和供水、供热、供气等公用公益事业市场化改革稳步推进。农村产权制度改革试点进展顺利，集体林权制度改革全面铺开。政府机构改革方案初步形成，开发区管理体制进一步理顺。推进科技创新。实施重大科技攻关项目50个，专利申请5200件，实现高新技术产业产值1660亿元，增长23%，科技进步对经济增长的贡献率提高到51%，我市被国家确定为知识产权工作示范城市和首批创新型试点城市。推进管理创新。重大项目、重大工程建设市场化进程加快，地铁建设、棚户区改造、水资源保护开发等重大市政项目实行了公司化运作。组建哈创投公司，以市场化、资本化运作方式支持投资创业。棚户区改造注重政策集成创新，“捆绑式开发”、“拆一还一”、“预约式拆迁”、“股折租”等政策发挥效力。进一步改进工作方式，领导包项目、包企业，机关干部下厂矿、进农村，实行季统计总结、月督查通报、周调度协调的项目建设制度和帮扶、定点联系企业制度，做到第一时间掌握情况、解决问题。推动银企对接，帮助企业克服和缓解融资难、贷款难。3户企业成功上市，使近三年新增境内外上市企业达到7户。我市获得“中国最具发展潜力金融生态城市”称号。大幅缩短建设项目审批时限，进入“绿色通道”的省市重点工程和哈大齐工业走廊、棚户区改造、市领导包保项目审批时限缩短到22天。

　　——加大民生工作力度，城乡人民生活水平获得新提高。拓展就业容量，力保民生之本。建立了1000万元创业扶持专项资金，发放小额担保贷款9226万元、改制企业捆绑式贷款1400万元，免费培训农民工33.8万人。加密社会“安全网”。失业、养老、医疗、工伤、生育保险覆盖面进一步扩大，为35.1万名低保对象发放低保金4.7亿元，为44万名困难群众和受灾群众发放救助金4.5亿元，整合建成敬老服务中心20所。改善群众居住条件。棚户区拆迁面积达173.1万平方米，建成回迁房127.8万平方米。1.6万棚改户喜迁新居，新增廉租住房受益户4412户，分别完成年计划的160%和220.6%。竣工经济适用住房171.97万平方米，户均货币补贴标准由3万元提高到4万元。为7500户职工家庭发放公积金贷款19.5亿元。拆除农村泥草房245.9万平方米，惠及农村居民4.17万户。统筹发展社会事业。170所中小学校达到标准化，35所城乡职业学校实现教育资源共享，10.2万名贫困学生获得资助，6万名农民工子女就学得到妥善安置。改造乡（镇）卫生院20所，600家村卫生所实现规范化管理，新农合参合率达到97.6%。启动了11类24项基本和重大公共卫生服务均等化项目，甲型H1N1流感等暴发性疫情得到有效控制。人口自然增长率控制在3.49‰以内。新建社区公益用房60处、社区图书分馆10个、乡（镇）综合文化站40个、农家书屋362个、群众文化辅导基地6个。21个项目列入第二批省级非物质文化遗产名录。完成有线电视数字化双向整转终端50万个，实现广播电视村村通。圆满完成第24届“大冬会”承办任务，实现参赛运动员总数和获金牌、奖牌数三个第一。妇女儿童、新闻出版、民族宗教、外事侨务、政府法制、人事、统计、审计、双拥、老龄、气象、人防、地震、档案、国防和预备役、地方志、残疾人等工作也取得新的进步。

　　各位代表，在应对危机过程中，我们更加深切地感到，省委、省政府和市委的坚强领导，市人大代表、政协委员及各界人士的鼎力支持，广大市民的辛勤努力和中省直驻哈企事业单位以及社会各界、海内外友好人士的积极帮助，比以往任何时候都弥足珍贵。在此，我代表市人民政府，向所有参与、支持和关注哈尔滨发展的同志们、朋友们，表示崇高的敬意和衷心的感谢！

　　我市经济社会发展中还存在一些突出矛盾和问题：经济总量较小、发展相对滞后是我市的主要矛盾，加快发展的压力还很大；大产业、大载体、大企业、大项目发展步伐还不够快，支撑力、拉动力还不够强，结构调整的任务还十分艰巨；县域经济偏弱，一些县（市）缺乏支柱产业和骨干龙头企业，富民强县的任务依然繁重；民生改善状况还不尽如人意，社会保障覆盖面还不广、标准还不高，改善民生的群众诉求还很热切；重特大交通事故等危及人民生命财产安全的责任事故没有得到根本遏制，确保公共安全的基层基础工作还比较薄弱；影响城市卫生、交通、服务环境的热点难点问题还很多，提高各级政府公共服务和社会管理水平的任务还十分迫切；一些政府工作人员精神不振、作风不实，不作为、乱作为，甚至为政不廉的现象还不同程度地存在，政府自身作风建设和效能建设依然紧迫。

　　2010年主要任务

　　今年，我们将按照“超越自我、再塑形象、奋起追赶、努力晋位，把哈尔滨建设成为现代大都市”的目标要求，以建设工业大城、科技新城、北国水城、文化名城、商贸都城为发展战略定位，全面实施“北跃、南拓、中兴、强县”发展战略。新的发展目标和战略将掀开哈尔滨思想大解放、经济大发展的新篇章。“一江居中、两岸繁荣”是城市未来发展的战略总纲。在松花江沿岸打造原生态的“万顷松江湿地、百里生态长廊”，并以松花江为中轴线，推动发展空间向两翼更广阔领域展开，构建大哈尔滨的发展格局。“北跃”就是以加快建设北国水城、科技创新城为战略支点，打造集科技、生态、文化于一体的现代新城区。“南拓”就是依托哈南地缘优势、产业基础，打造新型工业化集聚区，使之成为我市实现跨越晋位的强大引擎。 “中兴”就是用三年左右时间，搞好老城区路网改造、棚户区拆迁改造、地下管网和相应基础设施改造，环境治理和市容市貌建设，力促老城“弱势变优势、难点变靓点、旧貌换新颜”。“强县”就是加速提升县域经济实力，努力消除城乡“二元”结构，加快城乡一体化步伐。实施“北跃、南拓、中兴、强县”战略，是哈尔滨城市发展又一次历史性战略性突破，充分体现了省委、省政府对哈尔滨好发展快发展大发展的总体要求，充分反映了全市人民期盼好发展快发展大发展的强烈意愿，鼓舞人心，催人奋进，必将推动哈尔滨在新的起点上，赶超跨越，加速前进！

　　为全面打好、打胜起步之年第一仗，市政府工作总的要求是：坚持以科学发展观为指导，全面贯彻中央和省委经济工作会议、省委省政府专题会议及市委十二届八次全会精神，按照“超越自我、再塑形象、奋起追赶、努力晋位，把哈尔滨建设成为现代大都市”的要求，实施“北跃、南拓、中兴、强县”战略，调结构、上项目、靓环境、保民生、促稳定，为实现全市发展的总体目标奠定坚实基础。经济社会发展主要预期目标是：地区生产总值增长14%；规模以上工业增加值增长16%；地方财政一般预算收入增长16%；全社会固定资产投资增长40%以上；社会消费品零售总额增长17%左右；居民消费价格指数控制在103左右；市属外贸进出口总额增长15%；实际直接利用外资额增长10%；国内经济合作资金实际到位额增长30%以上；城市居民人均可支配收入增长10%左右；农村居民人均纯收入增长10%左右；城镇登记失业率控制在4.3%以内；人口自然增长率控制在4.5‰以内；万元生产总值综合能耗降低5%；水、气主要污染物在2005年基础上累计分别削减16%和11%。为全面完成预期目标，确保发展战略实施开好局、起好步，重点抓好八项工作：

　　（一）以哈南工业新城建设为重点，着力壮大工业经济实力

　　加快发展工业园区。以建设国际现代生态工业新城区为发展方向，高起点、高标准搞好哈南工业新城规划设计。依托平房和开发区现有工业基础，优化整合园区资源，完善功能配套，吸引优势企业和各种要素资源向园区集聚。启动哈南工业新城20.4平方公里起步区建设，力促一汽通用轻型车厂等100个在建项目竣工投产、中小企业创业园等100个新建项目开工建设、韩国工业园等100个协议项目尽快签约。加快哈大齐工业走廊哈尔滨段建设，突出抓好呼兰河工业核心示范区和宾西开发区建设，新增入区项目40个，完成投资超百亿元。

　　突出抓好重大工业项目。开展“项目建设提速年”活动，建立健全领导包保、通报监督、效能监察等项目推进机制，确保大项目建设全面提速。抓紧谋划和储备一批产业链长、拉动力强、技术含量高的工业大项目。抓好哈飞纯电动轿车、小型燃气轮机、超高压电力变压器等一批大项目建设，力促100个投资亿元以上、10个投资10亿元以上项目加快实施，50个在建项目竣工达产，完成工业投资680亿元，新增销售收入过100亿元。

　　重点培育龙头企业。着力培育一批主业突出、带动作用明显、拥有自主知识产权和知名品牌的大企业、大集团。支持企业通过兼并、重组、配套等方式加强合作，推进哈飞汽车、轻型车等企业国际合作进程。加大规模企业培育力度，力争新增规模以上工业企业100户。实施品牌战略，引导省级品牌向中国名牌、国家级驰名商标提升，引导拥有国家级自主品牌的企业向国际高端市场拓展，提高企业核心竞争力。

　　（二）实施“强县”战略，促进县域经济加快发展

　　以富民强县为核心，深入谋划县域经济发展。认真贯彻《关于实施强县战略的指导意见》，科学谋划县域经济实现新突破的新思路，加快推进城乡一体化、县域工业化、农村城镇化和农业产业化进程，努力形成强县率先突破、中等县迅速崛起、弱县加速追赶的新局面，尽快使更多县（市）进入全省、全国强县行列。

　　以农业基础设施建设为重点，持续增强农业综合生产能力。加强农田水利建设，推进亚布力等大中型水利枢纽、友谊等大灌区续建配套和节水改造工程，完成病险水库加固任务，农田水利设施保障率达到27%。加强高标准农田建设，重点实施水稻玉米双百万高产创建、旱作节水农业、中低产田改造、测土施肥、深松整地等工程，粮食产能稳定在220亿斤以上。抓好“菜篮子”工程，打造国家蔬菜标准园7个。加快水稻、玉米生产机械化进程，农机田间作业综合机械化程度达到80%。实施300万吨奶、200万头肉牛、1000万头猪、1亿只肉蛋鸡战略工程，标准化养殖场（户）增加到210 个，规模化养殖比重达到68%，畜牧业占农林牧渔业总产值的比重达到47%。加强气象灾害预报预警。强化农产品标识和质量监管追溯体系建设，市区主要农产品检测检疫实现全覆盖。

　　以推进农业产业化经营为重点，着力提升县域工业化水平。坚持工业化强县、产业化兴县，做大做强龙头企业，新增规模以上企业18家，参与产业化经营农户达到91万户。进一步明确工业园区功能定位，促进县域工业向特色园区集聚。把食品工业作为县域工业发展的主攻方向，重点建设英联食品酵母等10个农畜产品加工大项目。加快发展稻米、乳品等10大产业带，拉长农畜产品加工产业链，引导加工、流通、储运企业向优势产区集聚。加强农产品流通网络建设，增强供销合作社服务功能。打造绿色食品知名品牌，绿色、无公害、有机食品加工量和产值分别达到400万吨和120亿元。

　　以小城镇和新农村建设为重点，大力推进城镇化进程。重点支持40个县城和中心镇道路、给排水、垃圾处理等配套工程和新型农民居住社区建设，打造一批辐射能力强、各具特色的工业强镇、商贸大镇、旅游名镇、文化古镇。改善农民居住条件，改造泥草房4万户、300万平方米。加快安全饮水工程建设，解决12.8万人饮水安全问题，自来水普及率、安全饮水率分别达到51.6%和61.4 %。推进依兰至七台河高速公路建设，加快哈肇、铁通省级干线公路改造，开工建设高等级农村公路1200公里，竣工通车1000公里。强化农村生态环境治理，实施工程造林、水土流失治理等项目7个，10县（市）全部达到生态示范区标准。

　　（三）以转变发展方式为主线，着力推进经济结构战略性调整

　　以培育战略性新兴产业为重点，带动工业结构整体优化升级。加快发展新能源、新材料、生物等战略性新兴产业，推进太阳能、天然气、沼气、生物质能等绿色能源技术的开发与综合利用，加快哈空调风电机组、核电常规岛国产化、天顺化工碳纤维、哈药集团生物医药基地等重点项目建设。打造新能源装备产业集群，重点推进核电、风电、水电装备等产业项目。用高新技术改造传统产业，大力提升装备制造、食品、医药、化工等优势产业的高端化水平。围绕工业化与信息化融合，实施企业信息化“百家示范、千家提升、万家应用”工程，培育信息化示范企业100户。

　　以科技创新城为重点，引领高新技术产业加快发展。抓住国家创新型试点城市机遇，强力推进省市科技创新城建设，起步区今年开工达到50%以上，完成创新园起步区6.9平方公里和产业园起步区2.6平方公里基础设施建设，两园项目建设用地达到150万平方米。实施科技创新工程，重点突破电站成套装备、大型燃气轮机、畜禽重大疫病疫苗、节能减排等关键核心技术。加快发展高新技术产业，推进哈飞航空零部件基地等国家高技术产业化项目，培育LED照明、混合动力汽车等高新技术产业链条，推动铝镁合金新材料、发电设备等国家特色高新技术产业基地和生物产业、民用航空等国家高技术产业基地集群发展，确保高新技术产业产值增长20%以上。

　　以生产性服务业为重点，促进现代服务业提速增效。发展壮大现代金融业。推进哈尔滨银行等金融机构增资扩股、扩大业务，支持符合条件的企业上市融资、再融资，与天津股权交易所合作筹建区域性股权交易市场。深化小额贷款公司试点，小额贷款公司数量力争达到60家以上。发展壮大现代物流业。以建设区域性物流结点城市为目标，继续推进龙运等物流园区建设，吸引第三方物流企业来哈创业，加快公路、铁路、航空、航运四大物流港和物流公共信息平台建设。发展壮大旅游业。制定旅游业发展战略性规划，抓好亚布力特色旅游城镇等重点项目建设，依托生态湿地等旅游资源，推出一批更具吸引力的景点景区和特色旅游线路。加强旅游市场管理，完善服务功能，全面激活旅游市场。发展壮大商贸流通业。进一步调整优化商业网点布局，加快都市商圈提档升级和专业批发市场建设，增强商贸业的集中度和辐射力。深入实施“万村千乡市场工程”，实现乡（镇）、村农家店全覆盖。继续落实促进消费的各项政策，保持汽车、农机、家电等大宗商品的旺销势头。

　　以节能减排为重点，推动低碳经济发展。培育低碳产业，倡导低碳生活，探索开展碳交易、编制碳预算、追踪碳足迹。推进节能减排，着力抓好哈三电厂60万吨机组脱硫等8个减排项目，启动重点用能企业能源审计试点工作，加快建设龙能燃气公司生物质能综合利用等10个项目，工业固体废物综合利用率达到75%，规模以上工业企业万元增加值综合能耗下降5%以上。推进5个循环经济示范项目、2个示范园区建设。

　　（四）围绕塑造现代大都市形象，着力抓好老城区改造和新城区开发

　　加快构建大哈尔滨格局。以松花江为纽带和主轴，大手笔规划哈尔滨未来发展的宏伟蓝图，推动城市发展空间向北跨越、向南拓展、向县域延伸，塑造“万顷松江湿地、百里生态长廊”纵贯城市中央，松花江南岸欧陆风情与北岸现代特色北国水城相得益彰，科技创新城、工业新城拔地而起的现代大都市崭新形象。

　　加快跨江交通设施建设。松浦大桥建成通车，三环西桥确保进度，启动松花江公路大桥扩宽工程，配合铁路部门完成新滨洲桥拆迁征地、滨北公铁两用桥启动建设，为跨江发展创造先决条件。

　　加快北国水城建设。围绕打造原生态万顷松江湿地、构建松花江至呼兰河区域“两纵、四横、十八湖”北国水城水系目标，实施江北渠系开挖和提水泵站工程建设，水系建设骨干工程完成30%，同步实施超级堤防和沿江景观大道建设。完成江北文化中心设计招标并适时开工建设。突出文化、娱乐、体育休闲和冰雪文化功能定位，启动太阳岛核心地带建设。

　　加快群力和哈西新区开发。群力新区重点推进顾乡堤改线——友谊路延伸和滨江景观路建设，完成齿轮路以东区域道路和群力湿地公园等5大公园建设。哈西新区重点启动西客站两个站前广场、长途客运总站、公交换乘中心、7条区域道路和5座桥梁建设。

　　加快交通基础设施建设。推进地铁一号线一期工程，完成15座车站主体和5个区间隧道建设，启动地铁至哈西客站联络线工程和地铁环线前期工作。继续实施老城区路网结构升级改造。打通长江路、保健路、铁路街等10条断头路、卡脖路，调整完善地下管网。对省政府广场、霁虹桥等重要交通节点进行综合改造。新建各类桥涵57座、过街天桥24座，维修加固城区桥梁19座。新建和改造道路151条，维修翻浆破损道路326条（段），城市道路完好率达到90%以上。启动三环路江南段工程，建设机场迎宾路、学府路2座互通式立交桥和群力高架桥，改造老机场路等5条城市出口道路。

　　加快生态环境建设。治理松花江水污染，综合整治何家沟、信义沟和马家沟郊区段，实施“三沟”清水水源工程，打造清水常流的内河生态景观线，确保松花江干流出境水质达到三类、支流水质有所改善，全市污水集中处理能力达到150万吨/日，实现市区污水集中收集和处理。加快实施大绿化工程，植树100万株，新增绿地800公顷，绿化覆盖率和绿地率分别提高到38%和34%。加快道里集中供热三期等重点热源工程改扩建，拆除污染严重小锅炉100台，治理超标炉窑200座，削减烟尘排放量900吨。启动建筑垃圾综合处理厂、餐厨废弃油脂综合处理厂、粪便无害化处理场建设，提高垃圾和粪便处理无害化、减量化和资源化水平。打击企业违法排污，治理机动车尾气和噪声污染，清查危险废物和放射源，有效解决居民区噪声扰民问题。

　　加快干净、文明、有序城市建设。深化环卫清扫保洁和清冰雪作业市场化改革，抓好沿江沿河、城乡结合部和背街背巷环境卫生治理，严厉打击私卸乱倒等行为。加快整治城区铁路沿线环境，拆除环线30米范围内危旧房。全面清理户外牌匾广告，打造示范街路300条（段）。加大对流动商贩、马路市场和摊区的整治，市场摊区总量压缩到120个。强化建筑垃圾封闭运行管理，严格查处私建滥建行为。改善居民生活环境，改造建设主题公园5个、市民休闲健身广场50个、居民庭院500个、环保宜居社区50个，对城区内40万平方米裸露地实施覆盖。发挥街道办事处在城市管理中的基础作用，强化“门前四包”监督检查。加快数字城管建设，重点完成43个街道办事处三级网络系统连接。加强交通环境整治，严厉打击非法营运，重点整治火车站等窗口地区客运秩序，取缔大专院校等周边非法营运集结点，辟建停车泊位1.2万个。建设城市景观路，对会展旅游线路沿途破旧房屋实施拆除整饰，将中山路、机场迎宾路改造成标志性景观大道。整治沿江城区段环境，美化亮化部分公园、广场、建筑和友谊路、经纬街等78条主干街路以及火车站、霁虹广场等20处景观节点，真正让哈尔滨美起来、靓起来。

　　（五）全方位深化改革、扩大开放，着力增强经济发展的动力与活力

　　继续推进重点领域和关键环节改革。深化国有企业改革，积极推进哈轴集团等重点企业战略重组，健全公司法人治理结构、投资风险控制机制和内部监督管理机制。继续推进厂办大集体改革。深化农村各项改革，全面推进农村产权、集体林权制度改革；开展农村合作金融组织试点，活化农村资本40亿元；提高农民组织化程度，专业合作社发展到2000个，示范社增加到100个；积极化解乡村债务。继续加快推进财政、投融资以及公共卫生、公共交通、公共医疗和供水、供热、供气等公用公益事业市场化改革。深化政府机构改革，在人力资源、社会保障等6大领域、10个部门推行大部制，将政府机构由49个撤并为42个。

　　发展壮大非公有制经济。完善促进非公有制经济发展的体制机制，着力营造公平竞争的市场环境，凡是国家法律法规没有明文禁止的领域都允许民间资本进入。落实鼓励民间资本进入基础设施建设、公用事业、金融服务、社会事业等领域的相关政策，最大限度地保护民间投资的合法权益。强化中小企业发展专项资金、政策性信用担保等作用，积极争取商业银行的信贷支持，切实解决中小企业融资难问题。大力实施中小企业创业成长工程，新建市级以上中小企业创业基地3个，创办中小企业5000户，确保非公有制经济发展速度与质量明显高于全市平均发展水平。

　　努力扩大对外开放。着力调整出口商品结构，重点支持具有自主知识产权和自主品牌商品出口。大范围开拓国际市场，重点发展与中东、非洲、东盟等新兴市场的贸易。大规模培育骨干出口企业，鼓励有实力的企业走出去，重点发展大宗贸易。完成服务外包核心园区建设，力争软件服务外包收入增长30%以上。围绕对俄经贸适应性战略调整，加快对俄出口加工区建设，鼓励技术引进和双向投资，打造全省对俄进出口商品集散中心。围绕科技创新城、工业新城、老城改造、产业园区建设，谋划一批产业和区域开发项目，推广商业地产、集群式招商模式，利用“哈洽会”等经贸平台，扩大招商引资，力争使用外资规模和质量实现新跨跃。继续扩大与国内发达地区的经济合作，力争项目签约和引进资金实现新突破。

　　（六）全心全意改善和保障民生，着力让改革发展成果最大限度惠及群众

　　持续抓好惠民行动。公共财政继续向民生倾斜，全年安排改善办学条件、扩大公共服务等10大方面、60个惠民项目。

　　加快推进以棚户区改造为重点的保障性住房建设。抓住国家支持棚户区改造的有利时机，将改造范围外延至绕城高速以内，围绕三年基本完成现有棚户区1134万平方米改造目标，重点实施中华巴洛克历史文化街区保护改造、“三马”地区综合改造、城建重点项目关联棚户区改造、棚改项目回迁和保障性住房建设5项工程，今年拆迁改造345万平方米，建设总规模465万平方米，回迁1万户以上。做好保障性住房建设试点工作，规范利用住房公积金闲置资金。新增廉租住房受益户3000户，新建经济适用住房120万平方米，对1000户困难家庭实行购房货币补贴。棚户区改造和保障性住房建设是一场硬仗，也是一项最大的惠民工程，无论困难有多大，我们都要扭住不放，办得实而又实、好上加好。

　　促进创业带动就业。推进国家级创业型城市建设，全市创业活动指数达20%，带动就业比例达1：6,新增创业企业1.5万户、个体工商户3万户，新增企业存续1年以上的比例达到70%。加快大中专毕业生就业实训基地、农民工就业援助中心和工伤康复中心筹建步伐。强化针对就业困难人员特别是零就业家庭、高校毕业生和农民工群体的就业援助服务。完善政府购买培训成果机制，实行就业需求导向型培训。全市乡（镇）、村劳动保障平台建设力争实现全覆盖。深入实施百万农民工技能培训工程和“一十百千”农民增收计划，培训农民工20万人，转移农村劳动力128万人。

　　健全社会保障体系。落实社会保险扩面征缴和被征地农民基本养老保险政策，完善扩面责任目标体系，积极推进新型农村社会养老保险试点工作。努力探索将企事业长期临时工和乡镇企业职工纳入养老保险统筹办法，统筹解决关闭破产企业和困难企业退休人员参加医疗保险问题。提高城镇职工医疗支付限额，降低乙类药品自付比例，扩大医疗保险统筹金支付范围，支付比例达到75%以上。

　　完善城乡社会救助体系。加快社会救助能力建设，整合社会救助资源，开通社会救助网络系统。建立申请低保待遇人员收入比对机制，统筹做好城乡低保提标扩面及动态管理工作，城乡低保标准平均提高10%。创建25个国家级标准减灾示范社区，完善自然灾害救助应急预案体系。普遍实行区、县（市）医疗救助“一站式”结算。大力发展社会办养老机构，提高居家养老服务水平。建成20所农村敬老服务中心，建设占地10万平方米、1000张床位的失能老人护养院。完成儿童福利院等工程建设。农村五保户集中供养率达到50%。完成40个社区公益用房示范项目建设，农村社区建设启动50%。完成残疾人工作示范城市建设各项任务。

　　加快教育事业发展。加大教育投入。促进城乡义务教育均衡发展，搞好新区配套学校和寄宿制学校规划建设。为义务教育阶段农村和城市低保家庭学生免费提供教科书，为6万名农村寄宿制学校学生、1000名特殊教育学校学生、3万名中等职业学校学生、1.3万名家庭困难农民工子女提供生活补助。规范中小学校办学行为。大力发展职业教育和继续教育，加快建设市轻工学校和市幼儿师范高等学校。办好学前教育和地方高等教育。推进教育信息化，逐步实现优质教育资源共享。

　　加快完善公共卫生体系。新农合参合率达到98%以上，人均筹资标准提高到150元。装备城市社区卫生服务机构60个。健全农村三级医疗卫生服务网络，建设规范化村卫生所600个。继续实施“城市万名医师支援农村卫生工程”。在30%的公办基层医疗卫生服务机构实行药品零差率销售。城乡居民健康档案建档率分别达到70%和30%。开展“健康哈尔滨”活动。推进卫生应急指挥系统建设，提高公共突发事件应急和救治能力。全力做好甲型H1N1流感、结核病、艾滋病等重大传染性疾病防治工作。

　　大力发展文化和体育事业。加快发展文化产业和群众性文化事业。推进博物馆、艺术馆、音乐厅等文化设施及群力文化产业示范区建设。促进国有文艺演出团体改革和演艺资源重组。办好第30届“哈夏音乐会”。深入开展全民健身活动，安装健身路径1300件（套）。

　　（七）全面加强社会管理，着力促进社会和谐稳定

　　强化精神文明建设。以创建全国文明城市为牵动，带动环保模范城市、卫生城市、园林城市、基础设施达标城市建设。深入实施提升市民文明素质3年培训计划，深化创建文明社区、文明村镇、文明行业、文明单位和军警民共建共育活动，加强“文明创建合作区”建设，不断探索“以城带乡、城乡共建”的新途径。

　　强化民主法制建设。自觉接受人大及其常委会的法律监督和工作监督，认真执行人大及其常委会的决议、决定，高效率、高质量地办理人大代表建议和政协委员提案。完善民主决策、依法决策、科学决策机制，坚持专家咨询、社会公示和听证制度。依法开展第三届社区居委会换届选举工作，大力推进农村村民自治和民主管理。加强社会管理和公共服务等方面的政府立法，完善与国家法律相配套的地方政府规章体系。完善行政复议制度运行机制，提高政府解决行政争议的公信力。推进“法律六进”活动，搞好“五五”普法检查验收。

　　强化维护社会稳定工作。深入开展“平安哈尔滨”创建和社会治安综合治理，推进“天眼工程”二期建设和信息化建设。严厉打击涉黑、涉恶暴力犯罪、经济犯罪和侵财犯罪。加强网络虚拟社会监管。强化隐蔽战线斗争。加强安全生产监督管理，强化隐患排查治理，严格责任追究，严防各类事故发生。畅通群众诉求表达渠道，建立健全以两级信访服务中心为载体的信访工作新机制和社会稳定风险评估机制，完善和落实领导干部处理群众来信和接待群众来访制度，有效解决群众反映的问题。健全社会矛盾调处机制，加大社会矛盾纠纷排查化解力度，提高处置突发性群体性事件的能力。

　　（八）狠抓政府自身建设，确保经济发展环境明显改善

　　牢固树立宗旨意识，坚决清除制约发展的思想障碍。开展“作风效能建设年”主题实践活动，教育广大公务员牢固树立全心全意为人民服务的宗旨，用市委十二届七次、八次全会精神统一思想、凝聚力量、引领发展、检验工作。把超越自我、从严治政的要求贯穿政府自身建设全过程，下最大决心、用更大力度，对执政理念、工作作风、管理机制等方面与跨越赶超要求不符合、不适应的问题进行彻底整改，在对照自查中认清差距，在弃旧图新中超越自我，以对党、对人民、对事业高度负责的精神，更好地担负起推动好发展快发展大发展的重任。

　　切实转变工作作风，大力提高政府执行力。面对空前繁重的攻坚任务和全市人民的热切期盼，我们必须加大工作力度，加快工作节奏，做到说了算、定了办、马上干，干就干好。围绕解决跨部门、跨层级推进工作难问题，建立有效的部门协调机制，杜绝相互掣肘、互为门槛、推诿扯皮等内耗现象。持续推进会议、文件、出国、临时机构和办公经费“五减半”，着力革除文山会海、繁文缛节等行政弊端，让广大公务员把主要精力用在谋发展、抓落实上。全面推行政府绩效管理，把发展目标和任务细化分解，层层对标签状，层层落实责任，层层考核奖惩，用严明的责任机制保障工作落实，用有力的约束机制推动自我超越。

　　继续推进流程再造，提高行政审批服务效率。按照“项目建设提速年”要求，大力解决审批环节多、耗时长、“体外循环”和法律规定之外附加条件等企业反映强烈的问题，推动行政审批大提速。严格执行审批限时办结、超时默许等承诺制度，尽快建立行政效能投诉受理、查办、惩处机制，着力解决不作为、慢作为、乱作为和执行决策变形走样等突出问题，在哈尔滨政府各系统掀起一场有错必纠、有责必究的问责“风暴”。

　　深入推进反腐倡廉，建设干净干事的公务员队伍。越是在大发展的时候，越要毫不放松地抓好廉政建设，教育引导广大公务员自觉追求正派政绩，牢固树立干净干事的廉洁观念。进一步完善权力运行监管机制，坚持用制度管权、按制度办事、靠制度管人，重拳整治损害群众利益的突出问题。启动廉政巡查制度，强化对重点领域和关键环节的监督，特别要加大对重大投资项目的跟踪审计和全程监察，以更强有力的手段为好发展快发展大发展保驾护航。严肃查处官商勾结、权钱交易等违纪违法案件，对任何腐败行为和腐败分子都严惩不贷，大力营造风清气正的政务环境。

　　各位代表，2010年是全面完成“十一五”规划、科学编制“十二五”规划的重要一年，也是实施“北跃、南拓、中兴、强县”发展战略的起步之年，责任重大，使命光荣。让我们在省委、省政府和市委的坚强领导下，解放思想，坚定信心，振奋精神，埋头苦干，奋力推进经济社会好发展快发展大发展，为把哈尔滨建设成为现代大都市而奋斗！
